# FibroScan compared to liver biopsy for accurately staging recurrent hepatic steatosis and fibrosis after transplantation for MASH

**DOI:** 10.1111/liv.16085

**Published:** 2024-09-03

**Authors:** Laura Martínez‐Arenas, Carmen Vinaixa, Isabel Conde, Sara Lorente, Fernando Díaz‐Fontenla, Patrice Marques, Judith Pérez‐Rojas, Eva Montalvá, Ângela Carvalho‐Gomes, Marina Berenguer

**Affiliations:** ^1^ Hepatology, Hepatobiliopancreatic Surgery and Transplant Laboratory Instituto de Investigación Sanitaria La Fe (IIS La Fe) Valencia Spain; ^2^ Department of Biotechnology Universitat Politècnica de València Valencia Spain; ^3^ Centro de Investigación Biomédica en Red de Enfermedades Hepáticas y Digestivas (CIBEREHD) Instituto de Salud Carlos III Madrid Spain; ^4^ Hepatology and Liver Transplantation Unit Hospital Universitario y Politécnico La Fe Valencia Spain; ^5^ Hepatology and Liver Transplantation Unit Hospital Clínico Universitario Lozano Blesa, Instituto de Investigación Sanitaria Aragón (IIS Aragón) Zaragoza Spain; ^6^ Liver Unit and Digestive Department Hospital General Universitario Gregorio Marañón Madrid Spain; ^7^ Department of Pathology Hospital Universitario y Politécnico La Fe Valencia Spain; ^8^ Hepatobiliopancreatic Surgery and Transplantation Unit Hospital Universitario y Politécnico La Fe Valencia Spain; ^9^ Department of Surgery Universitat de València Valencia Spain; ^10^ Department of Medicine Universitat de València Valencia Spain

**Keywords:** FibroScan, liver biopsy, liver transplantation, metabolic dysfunction‐associated steatotic liver disease, metabolic factors, recurrent fibrosis, recurrent steatosis

## Abstract

**Background and Aims:**

Metabolic dysfunction‐associated steatotic liver disease (MASLD) recurrence after liver transplantation (LT) seems unavoidable and gradual. We aimed to evaluate the diagnostic accuracy in the post‐LT setting of patients transplanted for metabolic dysfunction‐associated steatohepatitis (MASH) of recurrent hepatic steatosis and fibrosis identified with FibroScan, compared to biopsy findings.

**Methods:**

This prospective cohort study included adults transplanted for MASH between 2010 and 2022 in three LT centres in Spain who underwent FibroScan and biopsy at least 1‐year after LT.

**Results:**

In total, 44 patients transplanted for MASH after LT were included. The median time from LT to biopsy and FibroScan was 24.5 (interquartile range [IQR]:16–46) and 26.0 (IQR: 16.8–41.5) months, respectively. The median time between biopsy and FibroScan was 2.0 (IQR: 0–5) months. On FibroScan, significant steatosis was diagnosed in about half of the patients (*n* = 21, 47.7%), yet advanced fibrosis in only two cases (4.6%). On biopsy, a quarter of biopsied patients (*n* = 11, 25%) had a MASH diagnosis, two (4.6%) with significant fibrosis and one (2.3%) with cirrhosis. All patients with liver stiffness measurement (LSM) values <8 kPa (*n* = 35, 79.5%) had a fibrosis stage ≤F1 (negative predictive value = 100%). The combination of post‐LT hypertension (odds ratio [OR]: 12.0, 95% confidence interval [CI]: 1.8–80.4, *p* = .010) and post‐LT dyslipidaemia (OR: 7.9, 95% CI: 1.3–47.1, *p* = .024) with LSM (OR: 1.7, 95% CI: 1.1–2.8, *p* = .030) was independently associated with MASLD.

**Conclusions:**

Although biopsy remains the gold standard for detecting fibrosis, our results suggest that LSM values <8 kPa after LT for MASH are strongly correlated with absence of significant/advanced fibrosis.

**Abbreviations**
CAP: control attenuation parameter
CI: confidence interval
HCV: hepatitis C virus
IQR: interquartile range
LSM: liver stiffness measurement
LT: liver transplant
MASH: metabolic dysfunction‐associated steatohepatitis
MASLD: metabolic dysfunction‐associated steatotic liver disease
MS: metabolic syndrome
NPV: negative predictive value
OR: odds ratio
PPV: positive predictive value

**Key points**

While liver biopsy remains the gold standard method for detecting graft fibrosis, non‐invasive alternative methods are frequently used for the follow‐up of liver transplant recipients. To date, FibroScan has only been validated for detecting graft fibrosis in patients transplanted for hepatitis C virus. Here, we report that FibroScan could be an accurate tool to diagnose the absence of significant/advanced fibrosis in liver grafts among patients transplanted for metabolic dysfunction‐associated steatohepatitis cirrhosis, and specifically to screen for those at no need of biopsy.

## 1 BACKGROUND

Metabolic syndrome (MS) is a cluster of derangements commonly affecting liver transplant (LT) recipients,^1^, ^2^ with a prevalence of 39.5% in the first year post‐LT but increasing with the follow‐up.^3^, ^4^ The emergence of metabolic dysfunction‐associated steatotic liver disease (MASLD) as the main chronic liver disease,^5^ with a prevalence of 32.4% worldwide,^6^ and as the fastest growing indication for LT in its progressive form,^7^ further complicates this scenario. In fact, the prevalence of post‐LT MS could be as high as 90% among those transplanted for metabolic dysfunction‐associated steatohepatitis (MASH), given their poor pre‐LT metabolic background.^8^

Unfortunately, MASLD can develop in the transplanted allograft as de novo MASLD or recurrent MASLD.^9^ Actually, recurrent (up to 50% of cases) and de novo MASLD (up to 20% of cases) occur in up to 70% of all LT recipients 12 months after the procedure.^10^, ^11^ Genetic, metabolic and pharmacological factors are associated with both recurrent and de novo MASLD after LT.^11^ Furthermore, Vallin et al. showed that recurrent MASLD tends to develop earlier and with greater severity than de novo MASLD.^12^ Additionally, allograft MASH is more common in recipients with a pre‐LT MASH diagnosis.^13^

While most patients with MASLD can be effectively diagnosed using non‐invasive tests available in the clinical practice,^14^ liver biopsy remains the gold standard method for evaluating ballooning degeneration, lobular inflammation and fibrosis,^15^, ^16^ and is frequently required for patients who have a positive diagnosis of MASLD and are at high or indeterminate risk for MASH and advanced fibrosis.^14^

The diagnostic accuracy of control attenuation parameter (CAP) and liver stiffness measurement (LSM) in identifying steatosis and fibrosis, respectively, assessed histologically in the non‐transplant setting, is widely acknowledged.^17^, ^18^, ^19^, ^20^ In the transplant context, FibroScan has only been validated for detecting graft fibrosis in patients transplanted for hepatitis C virus (HCV).^21^, ^22^, ^23^ However, the accuracy of FibroScan findings needs further assessment among patients transplanted for other indications.^24^

Therefore, this work aimed to evaluate the diagnostic accuracy in the posttransplant setting of patients transplanted for MASH of recurrent hepatic steatosis and fibrosis identified with FibroScan, compared to liver biopsy findings, as well as identify metabolic and pharmacological factors that, combined with CAP and LSM, could improve the accuracy of FibroScan.

## 2 MATERIALS AND METHODS

### 2.1 Study design and population

This prospective cohort study included adult patients transplanted for MASH cirrhosis between 2010 and 2022 in three LT centres in Spain who underwent both liver biopsy and FibroScan at least 1‐year after LT. After an exhaustive review of the clinical charts, each of these included individuals had documented MASH diagnosis before undergoing transplantation, as evidenced by the pre‐LT presence of overweight/obesity and/or type 2 diabetes mellitus as well as dyslipidaemia, hypertension or steatosis in >5% of hepatocytes in a previous liver biopsy or in the explant, in the absence of risky alcohol intake (≥20 g for women or ≥30 g for men per day) and other well‐known causes of liver fat accumulation, such as drugs with a steatogenic potential, Wilson's disease and HCV genotype 3.^25^, ^26^, ^27^, ^28^ Subjects transplanted for MASH‐associated hepatocellular carcinoma and cryptogenic cirrhosis with MS were also included in the study. We excluded patients under the age of 18 years, those positive for human immunodeficiency virus infection, those with previous or combined LT, except simultaneous liver–kidney transplant, and those with post‐LT potential factors that could aggravate fibrosis lesions, such as cellular rejection or biliary obstruction. Protocol biopsies were obtained at 1‐, 3‐ and 5‐year post‐LT, if possible, across the three centres. While the last biopsy and/or FibroScan were used for the primary analysis, a secondary analysis was done, including a subgroup of patients who underwent more than one biopsy and/or FibroScan after LT.

### 2.2 Liver biopsy

Experienced physicians performed percutaneous liver biopsies using a 16 Fr semi‐automated trucut‐type BioPince needle. Liver specimen was formalin‐fixed and paraffin‐embedded. Four‐μm‐thick serial sections were stained. The minimal staining included haematoxylin and eosin, Picro Sirius red and Masson's trichrome. All biopsies were assessed by a pathologist experienced in liver diseases who was blinded to the FibroScan results and clinical data.

Liver biopsy was evaluated according to the SAF (steatosis, activity and fibrosis) score.^29^ Steatosis was assessed from 0 to 3 (S0: <5%; S1: 5%–33%, mild steatosis; S2: 34%–66%, moderate steatosis; S3: >67%, marked steatosis). Activity was graded from 0 to 4 (A0: A = 0, no activity; A1: A = 1, mild activity; A2: A = 2, moderate activity; A3: A ≥ 3, severe activity). Fibrosis was evaluated according to the MASH Clinical Research Network Scoring System on a five‐stage scale: F0 (no fibrosis), F1 (perisinusoidal or periportal fibrosis: 1A—mild, zone 3, perisinusoidal; 1B—moderate, zone 3, perisinusoidal; 1C—portal/periportal), F2 (perisinusoidal and portal/periportal fibrosis), F3 (bridging fibrosis) and F4 (cirrhosis).^30^

The histopathological algorithm followed was: MASLD was defined by the presence of steatosis in >5% of hepatocytes; MASH was defined by the presence, in addition, of a SAF activity score ≥2. Significant and advanced fibrosis were defined as a fibrosis stage ≥F2 and ≥F3, respectively.^10^ Patients with known liver diseases different from MASLD/MASH were excluded from the analysis.

### 2.3 FibroScan

Using the FibroScan 502 device (Echosens, Paris, France), experienced hepatologists performed FibroScan according to the manufacturer's guidelines, blinded to the liver biopsy results and clinical data. FibroScan was considered successful only when at least 10 valid readings were obtained and the interquartile range (IQR)‐to‐median ratio of the 10 readings was ≤.3.

Hepatic steatosis and fibrosis were assessed by CAP and LSM, respectively. According to the CAP value, patients were divided into three groups: low steatosis (<250 dB/m), intermediate steatosis (250–300 dB/m) and high steatosis (>300 dB/m).^31^ CAP value >275 dB/m was used to diagnose significant steatosis.^32^ According to the LSM value, the following fibrosis stages were defined: F0 (0–5.9 kPa), F1 (6.0–6.9 kPa), F2 (7.0–9.0 kPa), F3 (9.1–10.3 kPa) and F4 (≥10.4 kPa).^33^ LSM values <8 kPa and >12–15 kPa were used to rule out and rule in advanced fibrosis, respectively.^32^

### 2.4 Data collection

Data were prospectively gathered, which contained clinical and demographic information, and were compiled in a unified database for analysis. Variables recorded at LT time were age and gender. Variables recorded at biopsy and/or FibroScan time were diabetes mellitus, hypertension, dyslipidaemia, overweight/obesity, tacrolimus and prednisone use and chronic kidney dysfunction.

### 2.5 Statistical analysis

Data were analysed using R version 4.3.1 (2023 The R Foundation for Statistical Computing Platform) and GraphPad Prism version 10.2.3. Data were described using mean (standard deviation) or median (IQR) for continuous variables, and *n* (%) for categorical variables. Continuous variables were compared using either the T‐test or non‐parametric tests (Wilcoxon or Kruskal–Wallis test), and categorical variables were compared using either the chi‐squared or Fisher's exact tests when appropriate. Multiple logistic regression analyses were used to assess the factors independently associated with MASLD. Variables associated with the dependent variable at univariate analysis (probability threshold, *p*‐value < .1) were included in the multivariate regression models. A *p*‐value < .05 was considered as a statistical significance.

### 2.6 Ethics

This study was conducted following both the Declaration of Helsinki and the Declaration of Istanbul. The study protocol was approved by La Fe University and Polytechnic Hospital's local ethics committee (Comité de Ética de la Investigación con Medicamentos [CEIm] del Hospital Universitario y Politécnico La Fe, 26 February 2020, registration number: 2019/0124) and by the Spanish Agency of Medicine and Sanitary Products (Agencia Española de Medicamentos y Productos Sanitarios [AEMPS], 20 January 2020, reference: 54823DD7BB). A protocol agreement was signed by principal investigators of the other centres (La Fe University and Polytechnic Hospital is the coordinator centre in charge of data storage and analysis). Written informed consent was obtained from each participant. The paper was reviewed and approved by all authors before submission.

## 3 RESULTS

### 3.1 Study cohort

As shown in the eligibility flow chart (Figure S1), among 52 subjects transplanted for MASH from 2010 to 2022 who underwent both liver biopsy and FibroScan at a minimum of one‐year post‐LT, three of them were excluded for (i) acute cellular rejection (*n* = 1, 1.9%), and (ii) biliary obstruction (*n* = 2, 3.8%). From this 49‐patient cohort, five of them were excluded for (i) non‐MASLD known liver diseases—autoimmune hepatitis (*n* = 1, 2.0%) and chronic hepatitis of unknown origin (*n* = 2, 4.1%), and for (ii) failure to obtain 10 valid readings on FibroScan (*n* = 2, 4.1%). The final cohort of 44 patients was used for subsequent analysis. The main characteristics of our cohort are described in Table S1. A third of patients were women (*n* = 14, 31.8%). Mean age at LT time was 60.3 ± 6.1 years. Rates of diabetes, arterial hypertension, dyslipidaemia and overweight/obesity at biopsy/FibroScan time were 68.2% (*n* = 30), 68.2% (*n* = 30), 63.6% (*n* = 28) and 79.6% (*n* = 35), respectively. Tacrolimus and prednisone were used at biopsy/FibroScan time in 42 (99.5%) and three (6.8%) patients, respectively. About half of patients (*n* = 25, 56.8%) had chronic kidney dysfunction at biopsy/FibroScan time.

### 3.2 Histopathological features

**FIGURE 1:**
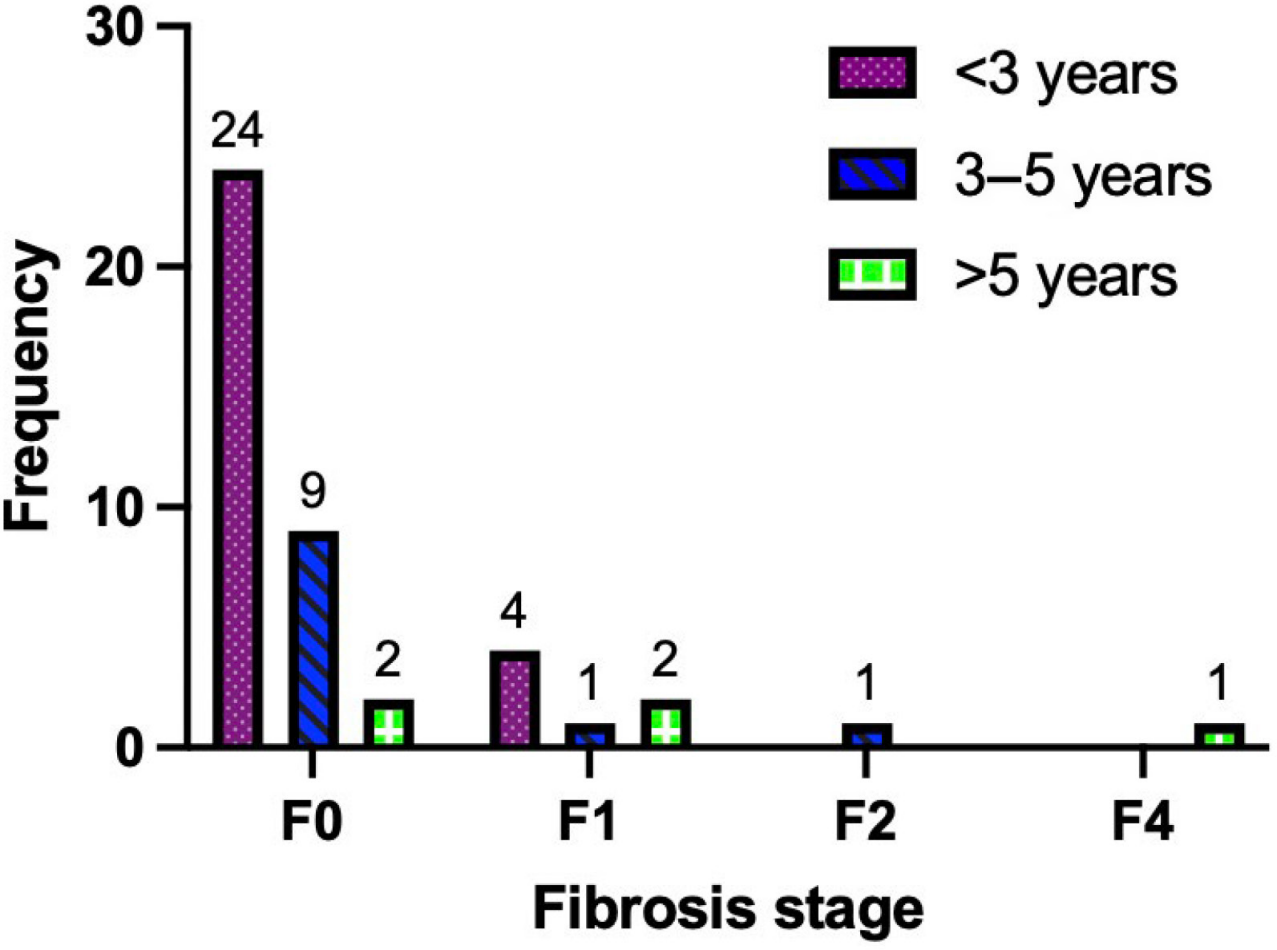
Fibrosis scores on liver biopsy over time from LT. LT, liver transplant.

Of 51 liver biopsies performed in the analysed subjects, 44 (86.3%) were conducted as a protocol biopsy. The histopathological features of MASLD are described in Table 1. The median time from LT to the last follow‐up biopsy was 24.5 (IQR: 16–46) months. About two‐thirds of the biopsied patients (*n* = 28, 63.6%) had a MASLD diagnosis, whereas a quarter (*n* = 11, 25%) had a MASH diagnosis, two (4.6%) with significant fibrosis and one (2.3%) with cirrhosis. The fibrosis scores over time from LT are described in Figure 1. The main characteristics of the cohort by MASLD/MASH diagnosis and fibrosis stage on biopsy are described in Tables S2 and S3, respectively. No significant differences were observed between the subgroup of patients who underwent protocol biopsies and those who underwent non‐protocol biopsies (Table 1). A seven‐patient subcohort underwent two serial biopsies after LT, with a median time of 13 (IQR: 11.5–18.5) months apart between them. All but one patient (*n* = 6, 85.7%) had a positive MASLD diagnosis in the first biopsy, with one (14.3%) having a MASH diagnosis in the second biopsy (Figure S2A). None of them had significant/advanced fibrosis, but two (28.6%) had a one‐stage rise of fibrosis stage (from F0 to F1), whereas one (14.3%) had a reversion of fibrosis stage (from F1 to F0) (Figure S2B).

**TABLE 1:** Histopathological features of MASLD.

	All cohort *n* = 44	Protocol biopsy cohort *n* = 39	Non‐protocol biopsy cohort *n* = 5	*p*
Time from LT (months), median (IQR)	24.5 (16–46)	23 (16–42)	46 (37–62)	.064
Steatosis degree, *n* (%)				
S0 (<5%)	16 (36.4%)	15 (38.5%)	1 (20.0%)	.691
S1 (5%–33%)	19 (43.2%)	16 (41.0%)	3 (60.0%)	
S2 (34%–66%)	3 (6.8%)	3 (7.7%)	N/A	
S3 (>67%)	6 (13.6%)	5 (12.8%)	1 (20.0%)	
Activity grade, *n* (%)				
A = 0 (none)	19 (43.2%)	18 (46.2%)	1 (20.0%)	.459
A = 1 (mild)	14 (31.8%)	12 (30.8%)	2 (40.0%)	
A = 2 (moderate)	6 (13.6%)	5 (12.8%)	1 (20.0%)	
A = 3 (severe)	3 (6.8%)	2 (5.1%)	1 (20.0%)	
A = 4 (very severe)	2 (4.6%)	2 (45.1%)	N/A	
Fibrosis stage, *n* (%)				
F0 (none)	35 (79.6%)	32 (82.1%)	3 (60.0%)	.147
F1a (mild, zone 3, perisinusoidal)	2 (4.6%)	2 (5.1%)	N/A	
F1b (moderate, zone 3, perisinusoidal)	N/A	N/A	N/A	
F1c (portal/periportal)	5 (11.4%)	4 (10.3%)	1 (20.0%)	
F2 (perisinusoidal and portal/periportal fibrosis)	1 (2.3%)	1 (2.6%)	N/A	
F3 (bridging fibrosis)	N/A	N/A	N/A	
F4 (cirrhosis)	1 (2.3%)	N/A	1 (20.0%)	
MASLD (≥S1)	28 (63.6%)	24 (61.5%)	4 (80.0%)	.638
MASH (≥S1 and A ≥2)	11 (25%)	9 (23.1%)	2 (40.0%)	.586
Significant fibrosis (≥F2)	2 (4.6%)	1 (2.6%)	1 (20.0%)	.217
Advanced fibrosis (≥F3)	1 (2.3%)	N/A	1 (20.0%)	.114

*Note*: Level of significance, *p*‐value < .05.

Abbreviations: IQR, interquartile range; LT, liver transplantation; MASH, metabolic dysfunction‐associated steatohepatitis; MASLD, metabolic dysfunction‐associated steatotic liver disease; N/A, not applicable.

### 3.3 FibroScan features

The analysed subjects underwent a total of 62 FibroScan measurements. The FibroScan features of MASLD are described in Table 2. The median time from LT to the last follow‐up FibroScan was 26.0 (IQR: 16.8–41.5) months. The median CAP and LSM values were 273 (IQR: 250.8–326.0) dB/m and 5.8 (IQR: 4.6–7.3) kPa (Figure S3), respectively, with significant steatosis diagnosed in about half of the patients (*n* = 21, 47.7%), yet advanced fibrosis in only two cases (4.6%). The main characteristics of the cohort by LSM and CAP on FibroScan measurement are described in Tables S4 and S5, respectively. No significant differences were noted between the subgroup of patients who underwent protocol and non‐protocol biopsies, nor between those FibroScan examinations with M and XL probes. (Table S6). An 18‐patient subcohort underwent two serial FibroScan measurements after LT, with a median time of 25.0 (IQR: 14.3–31.8) months apart between them. Significant steatosis was diagnosed in about one‐third of patients (*n* = 4, 36.4%, and *n* = 7, 38.9% in the first and second FibroScan examination, respectively) (Figure S4A) and advanced fibrosis in one case (5.6%) in both measurements, one case (5.6%) in the first examination, and one case (5.6%) in the second one (Figure S4B).

**TABLE 2:** FibroScan features of MASLD.

	All cohort *n* = 44	Protocol biopsy cohort *n* = 39	Non‐protocol biopsy cohort *n* = 5	*p*
Time from LT (months), median (IQR)	26 (16.8–41.5)	26 (16.5–40.0)	27 (26–119)	.306
LSM (kPa), median (IQR)	5.8 (4.6–7.3)	5.8 (4.7–7.3)	6.6 (4.4–8.8)	.725
CAP (dB/m), median (IQR)	273 (250.8–326.0)	273 (250.5–326.0)	268 (258.0–277.0)	.796
Steatosis degree, *n* (%)				
Low (<250 dB/m)	10 (22.7%)	9 (23.1%)	1 (20.0%)	.830
Intermediate (250–300 dB/m)	17 (38.6%)	14 (35.9%)	3 (60.0%)	
High (>300 dB/m)	17 (38.6%)	16 (41.0%)	1 (20.0%)	
Significant steatosis (>275 dB/m), *n* (%)	21 (47.7%)	19 (48.7%)	2 (40.0%)	.713
Fibrosis stage, *n* (%)				
F0 (0–5.9 kPa)	23 (52.3%)	21 (53.8%)	2 (40.0%)	.909
F1 (6.0–6.9 kPa)	6 (13.6%)	5 (12.8%)	1 (20.0%)	
F2 (7.0–9.0 kPa)	8 (18.2%)	7 (17.9%)	1 (20.0%)	
F3 (9.1–10.3 kPa)	N/A	N/A	N/A	
F4 (≥10.4 kPa)	7 (15.9%)	6 (15.4%)	1 (20.0%)	
Advanced fibrosis, *n* (%)				
Rule‐out (<8 kPa)	35 (79.6%)	32 (82.1%)	3 (60.0%)	.141
Rule‐in (>12–15 kPa)	2 (4.6%)	1 (2.6%)	1 (20.0%)	

*Note*: Level of significance, *p*‐value < .05.

Abbreviations: CAP, control attenuation parameter; IQR, interquartile range; LSM, liver stiffness measurement; LT, liver transplantation; MASLD, metabolic dysfunction‐associated steatotic liver disease; N/A, not applicable.

### 3.4 Liver biopsy versus FibroScan

**FIGURE 2:**
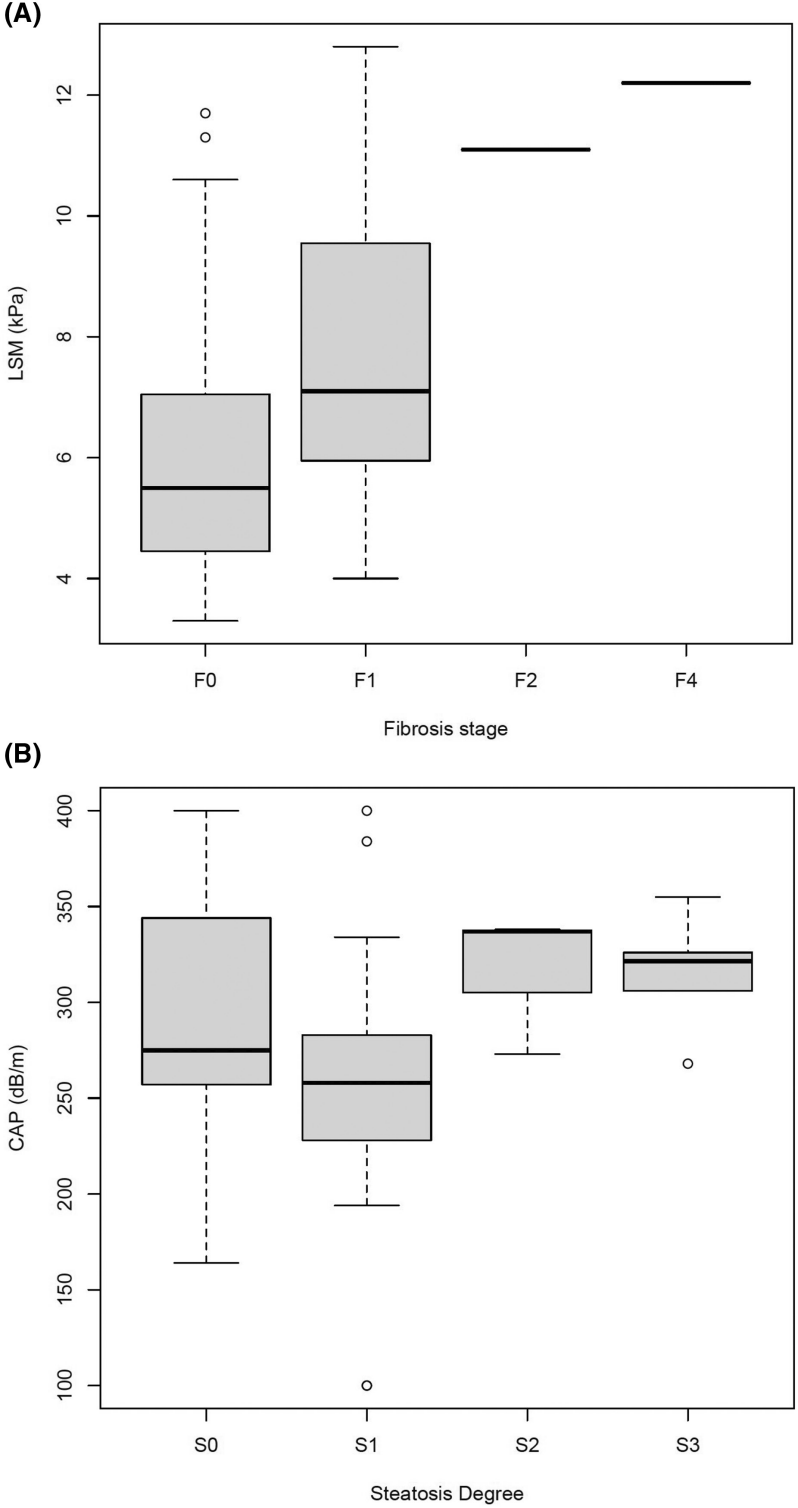
Boxplot of: (A) LSM on FibroScan and fibrosis stage on liver biopsy; (B) CAP on FibroScan and steatosis degree on liver biopsy. CAP, control attenuation parameter; LSM, liver stiffness measurement.

The median time between liver biopsy and FibroScan measurement was 2 (IQR: 0–5) months, with 35 (79.6%) patients having a lag time lower than 6 months between both techniques. A correlation was found between fibrosis stage on graft biopsy and elastographic values on FibroScan. All patients with LSM values <8 kPa (*n* = 35, 79.5%) had a fibrosis stage ≤F1 (negative predictive value [NPV] = 100%), whereas of those with LSM values ≥8 kPa (*n* = 9, 20.5%), only 2 (22.2%) had a fibrosis stage ≥F2 (positive predictive value [PPV] = 20%), with one (11.1%) case with cirrhosis (*p* = .038) (Table 3; Figure 2A). No significant differences were noted between the absence/presence of significant steatosis by FibroScan and steatosis degree on biopsy (NPV = 30% and PPV = 60%) (Table 4, Figure 2B). On multivariate analysis, LSM (odds ratio [OR]: 1.7, 95% confidence interval [CI]: 1.1‐2.8, *p* = .030), post‐LT hypertension (OR: 12.0, 95% CI: 1.8–80.4, *p* = .010) and post‐LT dyslipidaemia (OR: 7.9, 95% CI: 1.3–47.1, *p* = .024) were significantly associated with MASLD (Table 5).

**TABLE 3:** Correlation between LSM on FibroScan and fibrosis stage on liver biopsy.

	LSM <8 kPa *n* = 35	LSM ≥8 kPa *n* = 9	*p*
F0 versus ≥F1, *n* (%)	30 (85.7)	5 (55.6)	.068
F0–F1 versus ≥F2, *n* (%)	35 (100)	7 (77.8)	**.038**
F0–F3 versus F4, *n* (%)	35 (100)	8 (88.9)	.204

*Note*: Level of significance, *p*‐value < .05.

Abbreviation: LSM, liver stiffness measurement.

**TABLE 4:** Correlation between CAP on FibroScan and steatosis degree on liver biopsy.

	CAP ≤275 dB/m *n* = 23	CAP >275 dB/m *n* = 21	*p*
S0 versus ≥S1, *n* (%)	8 (34.8)	8 (38.1)	.820
S0–S1 versus ≥S2, *n* (%)	21 (91.3)	14 (66.7)	.064
S0–S2 versus S3, *n* (%)	22 (95.7)	16 (76.2)	.088

*Note*: Level of significance, *p*‐value < .05.

Abbreviation: CAP, control attenuation parameter.

**TABLE 5:** Univariate and multivariate analysis of factors associated with MASLD (defined as a steatosis degree >S1).

	Univariate analysis		Multivariate analysis	
	OR (95% CI)	*p*	OR (95% CI)	*p*
LSM (kPa)	1.5 (1.0–2.1)	.035	1.7 (1.1–2.8)	.030
Hypertension at bx/FS time	6.0 (1.5–23.6)	.012	12.0 (1.8–80.4)	.010
Dyslipidaemia at bx/FS time	3.9 (1.0–14.2)	.043	7.9 (1.3–47.1)	.024

*Note*: Level of significance, *p*‐value < .05.

Abbreviations: Bx, biopsy; CI, confidence interval; FS, FibroScan; MASLD, metabolic dysfunction‐associated steatotic liver disease; LSM, liver stiffness measurement; OR, odds ratio.

## 4 DISCUSSION

In this prospective study on a population of LT recipients for MASH, our results suggest that LSM values <8 kPa after LT for MASH are strongly correlated with the absence of significant/advanced fibrosis. Therefore, FibroScan could be an effective non‐invasive tool to stage liver fibrosis recurrence among patients transplanted for MASH cirrhosis, and specifically to screen for those at no need of biopsy. Additionally, the combination of post‐LT hypertension and post‐LT dyslipidaemia with LSM is independently associated with MASLD, which could improve the accuracy of FibroScan.

The biopsy data show recurrence rates of steatosis, MASH and advanced fibrosis of 63.6%, 25.0% and 2.3%, respectively. In a meta‐analysis including 17 studies involving 2378 patients, the incidence of recurrent MASLD and MASH was 82% and 38%, respectively, at more than 5 years after LT.^34^ Villeret et al. showed recurrence rates of steatosis, MASH and advanced fibrosis of 80.0%, 60.3% and 20.0%, respectively, at 5 years after LT, in a cohort including mainly protocol biopsies.^10^ Our study cohort shows a high proportion of protocol liver biopsies as well, yet the biopsies were performed earlier than in the literature (most in the first 2 years post‐LT), so our results may not be comparable to other studies with longer follow‐up. In fact, patients with biopsy >3 years after LT were those with significant/advanced fibrosis.

When comparing our data on liver biopsy versus FibroScan, the diagnostic accuracy of FibroScan in evaluating graft fibrosis correlates significantly with the absence of significant/advanced fibrosis when the recommended LSM cut‐off of 8 kPa for ruling out advanced fibrosis was applied.^32^ Actually, our study demonstrates a 100% NPV for FibroScan in identifying absence of fibrosis among our post‐LT patient cohort, such that significant/advanced fibrosis can be reliably rule out without the need for more invasive procedures, such as biopsies. By effectively identifying patients who do not require further diagnostic interventions, we can reduce their exposure to unnecessary risks and associated healthcare costs. Undoubtedly, the LT recipient is extremely complex, and some diagnoses, such as drug‐induced liver injury, may only be captured by liver biopsy.^35^, ^36^

Our study is consistent with the findings of Lutz et al. regarding FibroScan for a non‐invasive evaluation of graft fibrosis in 48 LT recipients transplanted for any indication compared with the protocol biopsy 1‐year after LT.^37^ Additionally, Lutz et al. suggested that FibroScan in combination with Doppler ultrasound should be implemented as non‐invasive hepatic tools in the evaluation and surveillance during the follow‐up after LT. In our study, the significant associations between LSM, post‐LT hypertension, post‐LT dyslipidaemia, and MASLD underscore the need for comprehensive cardiovascular risk management in post‐LT patients. Regular monitoring and control of blood pressure and lipid levels should be integrated into post‐transplant care protocols to reduce the risk of MASLD.

When comparing our data on liver biopsy and FibroScan for evaluating graft steatosis, no correlation was found between both techniques when the non‐consensual CAP cut‐off of 275 dB/m for diagnosing significant steatosis was applied^32^; consistent with the recommendation of using conventional ultrasound as the first‐line non‐invasive tool for the diagnosis of steatosis in clinical practice.^37^ Yet, the potential for steatosis to change substantially in 2–6 months between FibroScan and biopsy may impact the comparability of both techniques. Additionally, the patchy nature of steatosis is known to be a reason for both tests to be less accurate as well.

The main limitation of this study is that it includes a small number of patients with recurrent significant/advanced fibrosis, since in most patients (75%), liver biopsy and FibroScan were performed no longer than approximately 3.5 years from LT (most within 2 years), probably a too early follow‐up time in the development of graft fibrosis in the setting of MASH recurrence. Our findings are, in fact, more useful to rule out advanced fibrosis after LT; but unfortunately, we were unable to correlate histology data with the suggested LSM cut‐off of 12 kPa for ruling in advanced fibrosis.^32^

While a 100% NPV is highly valuable, it is important to acknowledge the context and limitations of this finding. The high NPV observed in our study may be influenced by the specific characteristics of our study population, including demographics, prevalence of MASLD and underlying conditions. Sex differences impact in the development of steatosis and fibrosis post‐LT.^38^ Our data are predominantly applicable to the older male cohort, yet the two cases with significant fibrosis were women. Female LT recipients, particularly those who are postmenopausal, are at a higher risk of developing steatosis and fibrosis compared to their male counterparts.^39^ Furthermore, our data are specific to the region and reflect a currently low prevalence of MASH. Given the lower risk and possibly different lifestyle of the region, the results may not be applicable to other geographic areas. Therefore, caution must be exercised when generalizing these results to broader or different patient populations. Additionally, the performance of the test in clinical practice may vary based on factors such as the prevalence of MASLD in different settings and the implementation of the test protocol.

Finally, the strongest predictors of recurrent/de novo MASLD are either donor‐related (genetic), and recipient‐related (metabolic and pharmacological).^11^ Although some genetic polymorphisms, namely PNPLA3, TM6SF2 and MBOAT7, represent critical determinants in the pathogenesis of liver steatosis and in the progression of liver damage and could be used in this diagnostic setting, very few studies have explored the role of their combination in MASLD diagnosis and in the prediction of evolving disease.^40^ While our current study did not specifically investigate the combination of genetic factors with CAP and LSM, we recognize the potential value of such an analysis. In addition, we could not also investigate the association of these factors with graft fibrosis because there were so few patients with advanced fibrosis.

In summary, while liver biopsy remains the gold standard method for detecting graft fibrosis in the post‐LT setting of patients transplanted for MASH, our current data suggest that the FibroScan might be an accurate tool to diagnose the absence of significant/advanced fibrosis in liver grafts due to MASH recurrence. We believe that our findings are relevant considering that, to date, FibroScan has only been validated for the follow‐up of HCV recurrence in the post‐transplant setting.

## AUTHOR CONTRIBUTIONS

Conceptualization: Marina Berenguer. Methodology: All authors. Data curation: Laura Martínez‐Arenas, Sara Lorente and Fernando Díaz‐Fontenla. Formal analysis: Laura Martínez‐Arenas. Writing—original draft: Laura Martínez‐Arenas. Writing—review and editing: All authors. Supervision: Ângela Carvalho‐Gomes and Marina Berenguer.

## FUNDING INFORMATION

This research was funded by the Instituto de Salud Carlos III and co‐funded by European Regional Development Fund ‘A way to make Europe’ (grant PI19/01360 to MB, grant INT20/00061 to MB, grant FI20/00033 to LMA, grant MV22/00053 to LMA, grant CD22/00045 to PM, grant PI23/00088 to MB, and grant INT24/00021 to MB), by the Generalitat Valenciana (grant AICO/2021/035 to MB, grant CIGE/2023/073 to PM, and grant CIPROM/2023/16 to MB), by the CIBER ‐Consorcio Centro de Investigación Biomédica en Red‐ [CB06/04/0065], Instituto de Salud Carlos III, Ministerio de Ciencia e Innovación and Unión Europea—European Regional Development Fund, and by the Spanish Society of Liver Transplantation (SETH, grant 2022/295 to MB). No sponsor had a role in the study design, the data collection, the analysis and interpretation of data, the writing of the paper or the decision to submit the article for publication.

## CONFLICT OF INTEREST STATEMENT

The authors declare no conflicts of interests related to this work.

## Supporting information


DATA S1:

## Data Availability

The data that support the findings of this study are available from the corresponding author upon reasonable request.
