# Supplementary material for: FibroScan compared to liver biopsy for accurately staging recurrent hepatic steatosis and fibrosis after transplantation for MASH
Source: Liver Int. 2024 Sep 3;44(12):3174–82. doi: 10.1111/liv.16085 (PMC11586891; doi:10.1111/liv.16085)
Supplement: Supplementary file 1 — DATA S1: [file LIV-44-3174-s001.docx]

**FibroScan compared to liver biopsy for accurately staging recurrent hepatic steatosis and fibrosis after transplantation for MASH**

Laura Martínez-Arenas, Carmen Vinaixa, Isabel Conde, Sara Lorente, Fernando Díaz-Fontenla, Patrice Marques, Judith Pérez-Rojas, Eva Montalvá, Ângela Carvalho-Gomes, Marina Berenguer

**Table of contents**

Fig. S1………….……………………………………………………………………………..2

Fig. S2…………….…………………………………………………………………………..3

Fig. S3…………….…………………………………………………………………………..4

Fig. S4……………………………………………………………………………………..….5

Table S1………………………………………………………………………………..……..6

Table S2………………………………………………………………………………..……..7

Table S3………………………………………………………………………………..……..8

Table S4………………………………………………………………………………..……..9

Table S5……………………………………………………………………...………..…....10

Table S6………………………………………………………………………………..…....11

Hospital B

n = 9 (17.3%)

Hospital C

n = 6 (11.5%)

Hospital A

n = 37 (71.2%)

**Analyzed subjects**

**n = 44**

**Included**

**n = 49**

51 biopsies

**Screening**

**n = 52**

Excluded:

- Acute cellular rejection: n = 1 (1.9%)

- Biliary obstruction: n = 2 (3.8%)

Excluded:

- Non-MASLD known liver diseases: n = 3 (6.1%)

- FibroScan failure: n = 2 (4.1%)

62 FibroScan measurements

**Fig. S1.** Patient eligibility flow chart.

a)


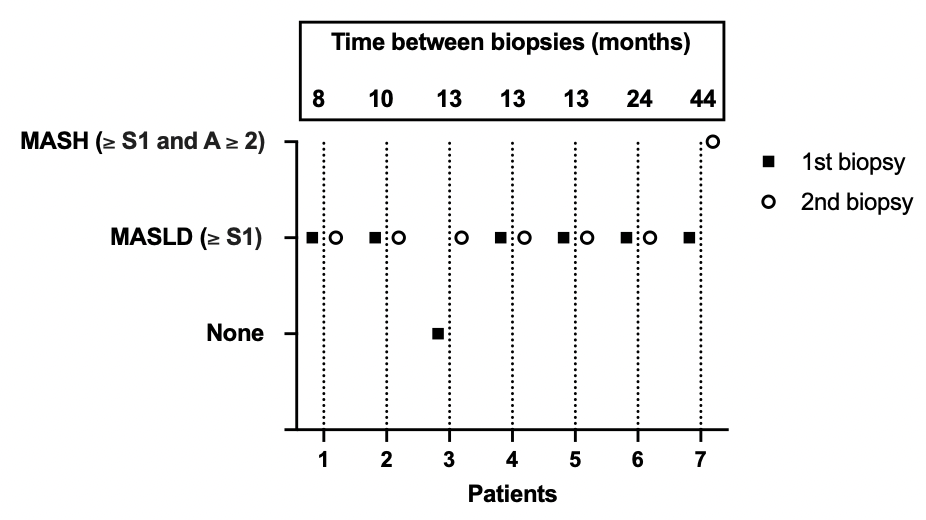


b)


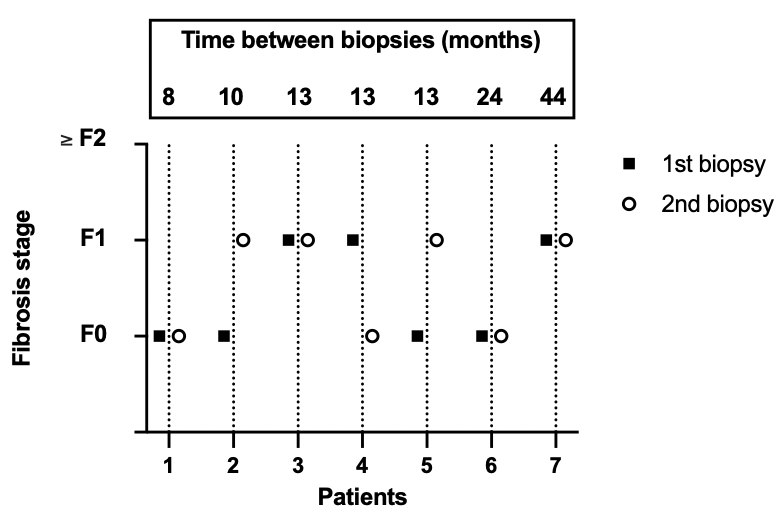


**Fig. S2.** Histopathological features of MASLD in the subcohort with serial biopsies (n=7): a) MASLD/MASH diagnoses in the first and second biopsy and the time between them; b) Fibrosis stage in the first and second biopsies and the time between them.

**
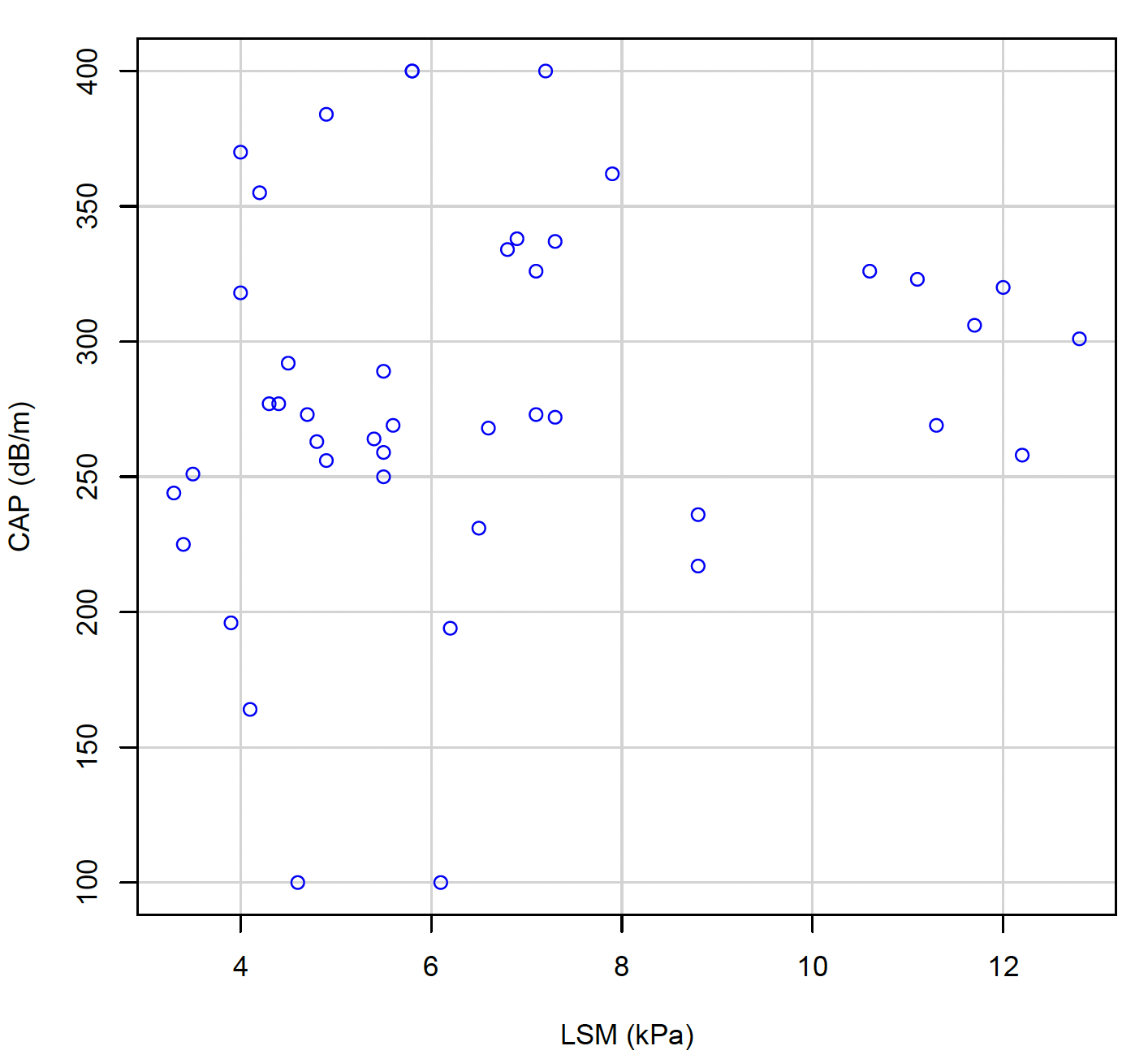
**

**Fig. S3.** Scatter plot of LSM and CAP values measured on FibroScan.

a)


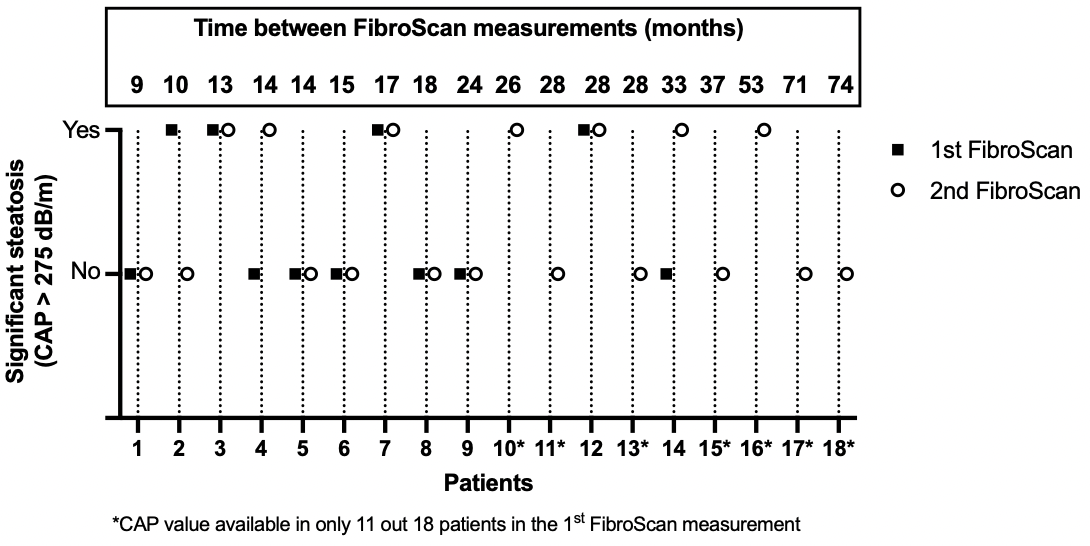


b)


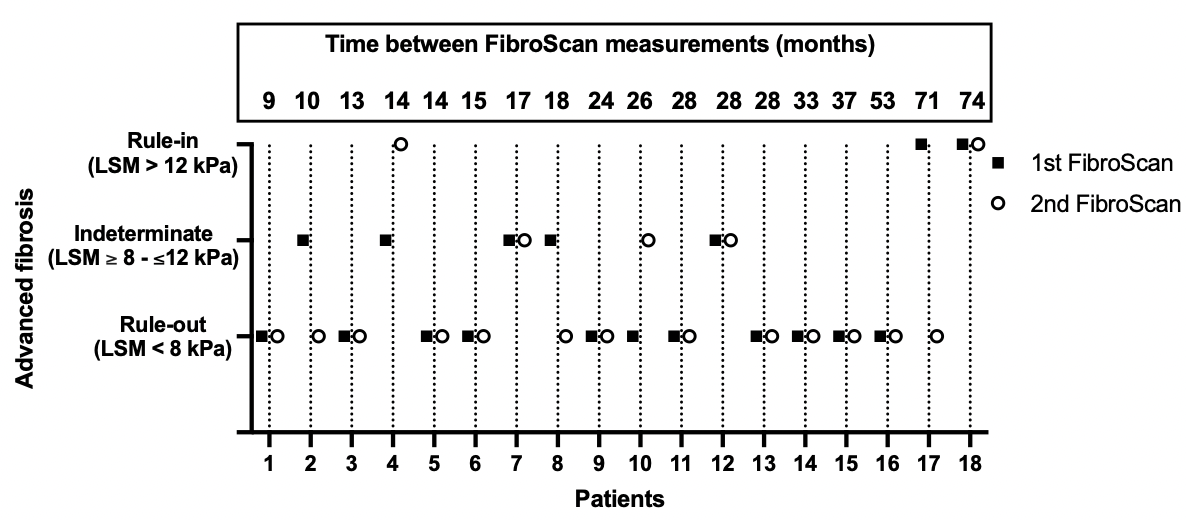


**Fig. S4.** FibroScan features of MASLD in the subcohort with serial FibroScan measurements (n=18): a) Significant steatosis diagnosis in the first and second FibroScan measurement and the time between them; b) Advanced fibrosis in the first and second FibroScan measurement and the time between them.

**Table S1.** Demographic and clinical characteristics of our cohort.

|  | Analyzed subjects  n=44 |
| --- | --- |
| Age at LT time, years, mean (SD) | 60.3 (6.1) |
| Gender, females, % (n) | 31.8 (14) |
| Components of MS at bx/FS time, % (n):  -Diabetes mellitus  -Hypertension  -Dyslipidemia  -Overweight/Obesity | 68.2 (30)  68.2 (30)  63.6 (28)  79.6 (35) |
| Tacrolimus use at bx/FS time, % (n) | 99.5 (42) |
| Prednisone use at bx/FS time, % (n) | 6.8 (3) |
| Chronic kidney dysfunction (glomerular filtration rate <70 ml/min) at bx/FS time, % (n) | 56.8 (25) |

Bx, biopsy; FS, FibroScan; LT, liver transplantation; MS, metabolic syndrome; SD, standard deviation

**Table S2.** Demographic and clinical characteristics by MASLD/MASH diagnosis on liver biopsy.

|  | Non-MASLD  n=16 | MASLD  n=17 | MASH  n=11 | *p* |
| --- | --- | --- | --- | --- |
| Age at LT time, years, mean (SD) | 60.2 (7.2) | 59.9 (6.1) | 61.2 (4.5) | 0.867 |
| Gender, females, % (n) | 25 (4) | 29.4 (5) | 45.5 (5) | 0.514 |
| Components of MS at bx/FS time,  % (n):  -Diabetes mellitus  -Hypertension  -Dyslipidemia  -Overweight/Obesity | 68.8 (11)  43.8 (7)  43.8 (7)  75.0 (12) | 58.8 (10)  82.4 (14)  64.7 (11)  82.4 (14) | 81.8 (9)  81.8 (9)  90.9 (10)  81.8 (9) | 0.442  **0.032**  **0.043**  0.897 |
| Tacrolimus use at bx/FS time, % (n) | 100 (16) | 94.1 (16) | 90.9 (10) | 0.713 |
| Prednisone use at bx/FS time, % (n) | 6.2 (1) | 5.9 (1) | 9.1 (1) | >0.999 |
| Chronic kidney dysfunction (glomerular filtration rate <70 ml/min) at bx/FS time, % (n) | 56.2 (9) | 52.9 (9) | 63.6 (7) | 0.854 |

Level of significance, *p*-value <0.05

Bx, biopsy; FS, FibroScan; LT, liver transplantation; MASH, metabolic dysfunction-associated steatohepatitis; MASLD, metabolic dysfunction-associated steatotic liver disease; MS, metabolic syndrome; N/A, not applicable; SD, standard deviation

**Table S3.** Demographic and clinical characteristics by fibrosis stage on liver biopsy.

|  | F0  n=35 | F1  n=7 | F2-F4  n=2 | *p* |
| --- | --- | --- | --- | --- |
| Age at LT time, years, mean (SD) | 59.5 (6.3) | 63.0 (4.9) | 64.5 (2.7) | 0.234 |
| Gender, females, % (n) | 22.9 (8) | 57.1 (4) | 100 (2) | **0.022** |
| Components of MS at bx/FS time,  % (n):  -Diabetes mellitus  -Hypertension  -Dyslipidemia  -Overweight/Obesity | 74.3 (26)  62.9 (22)  65.7 (23)  80.0 (28) | 42.9 (3)  85.7 (6)  57.1 (4)  85.7 (6) | 50.0 (1)  100 (2)  50.0 (1)  50.0 (1) | 0.226  0.355  0.851  0.568 |
| Tacrolimus use at bx/FS time, % (n) | 97.1 (34) | 100 (7) | 50.0 (1) | 0.112 |
| Prednisone use at bx/FS time, % (n) | 8.6 (3) | N/A | N/A | >0.999 |
| Chronic kidney dysfunction (glomerular filtration rate <70 ml/min) at bx/FS time, % (n) | 54.3 (19) | 57.1 (4) | 100 (2) | 0.602 |

Level of significance, *p*-value <0.05

Bx, biopsy; FS, FibroScan; LT, liver transplantation; MS, metabolic syndrome; N/A, not applicable; SD, standard deviation

**Table S4.** Demographic and clinical characteristics by LSM on FibroScan.

|  | LSM <8 kPa  n=35 | LSM ≥8 kPa  n=9 | *p* |
| --- | --- | --- | --- |
| Age at LT time, years, mean (SD) | 60.9 (6.3) | 58.2 (4.8) | 0.252 |
| Gender, females, % (n) | 34.3 (12) | 22.2 (2) | 0.695 |
| Components of MS at bx/FS time,  % (n):  -Diabetes mellitus  -Hypertension  -Dyslipidemia  -Overweight/Obesity | 71.4 (25)  65.7 (23)  60.0 (21)  85.7 (30) | 55.6 (5)  77.8 (7)  77.8 (7)  55.6 (5) | 0.434  0.695  0.450  0.068 |
| Tacrolimus use at bx/FS time, % (n) | 97.1 (34) | 88.9 (8) | 0.371 |
| Prednisone use at bx/FS time, % (n) | 5.7 (2) | 11.1 (1) | 0.567 |
| Chronic kidney dysfunction (glomerular filtration rate <70 ml/min) at bx/FS time, % (n) | 45.7 (16) | 100 (9) | **0.006** |

Level of significance, *p*-value <0.05

Bx, biopsy; FS, FibroScan; LSM, liver stiffness measurement; LT, liver transplantation; MS, metabolic syndrome; N/A, not applicable; SD, standard deviation

**Table S5.** Demographic and clinical characteristics by CAP on FibroScan.

|  | CAP ≤275 dB/m  n=23 | CAP >275 dB/m  n=21 | *p* |
| --- | --- | --- | --- |
| Age at LT time, years, mean (SD) | 59.6 (6.2) | 61.1 (6.0) | 0.432 |
| Gender, females, % (n) | 39.1 (9) | 23.8 (5) | 0.276 |
| Components of MS at bx/FS time,  % (n):  -Diabetes mellitus  -Hypertension  -Dyslipidemia  -Overweight/Obesity | 65.2 (15)  69.6 (16)  56.5 (13)  73.9 (17) | 71.4 (15)  66.7 (14)  71.4 (15)  85.7 (18) | 0.659  0.837  0.305  0.462 |
| Tacrolimus use at bx/FS time, % (n) | 100 (23) | 90.5 (19) | 0.222 |
| Prednisone use at bx/FS time, % (n) | 8.7 (2) | 4.8 (1) | >0.999 |
| Chronic kidney dysfunction (glomerular filtration rate <70 ml/min) at bx/FS time, % (n) | 60.9 (14) | 52.4 (11) | 0.570 |

Level of significance, *p*-value <0.05

Bx, biopsy; FS, FibroScan; CAP, control attenuation parameter; LT, liver transplantation; MS, metabolic syndrome; N/A, not applicable; SD, standard deviation

**Table S6.** FibroScan features of MASLD regarding the probe type.

|  | FibroScan with M probe  n=28 | FibroScan with XL probe  n=16 | *p* |
| --- | --- | --- | --- |
| Time from LT (months), median (IQR) | 25.5 (16.8-41.5) | 27.0 (22.0-42.0) | 0.385 |
| LSM (kPa), median (IQR) | 5.5 (4.3-7.4) | 6.0 (4.9-7.3) | 0.360 |
| CAP (dB/m), median (IQR) | 268.5 (242.0-321.5) | 284.5 (267.5-328.8) | 0.204 |
| Steatosis degree, n (%):  -Low (< 250 dB/m)  -Intermediate (250 – 300 dB/m)  -High (> 300 dB/m) | 8 (28.6%)  10 (35.7%)  10 (35.7%) | 2 (12.5%)  7 (43.8%)  7 (43.8%) | 0.484 |
| Significant steatosis (> 275 dB/m), n (%) | 12 (42.9%) | 9 (56.2%) | 0.392 |
| Fibrosis stage, n (%):  -F0 (0 – 5.9 kPa)  -F1 (6.0 – 6.9 kPa)  -F2 (7.0 – 9.0 kPa)  -F3 (9.1 – 10.3 kPa)  -F4 (≥ 10.4 kPa) | 15 (53.6%)  5 (17.9%)  4 (14.3%)  N/A  4 (14.3%) | 8 (50.0%)  1 (6.2%)  4 (25%)  N/A  3 (18.8%) | 0.691 |
| Advanced fibrosis, n (%):  -Rule-out (< 8 kPa)  -Rule-in (> 12 – 15 kPa) | 22 (78.6%)  N/A | 13 (81.2%)  2 (12.5%) | 0.120 |

Level of significance, *p*-value <0.05

CAP, control attenuation parameter; IQR, interquartile range; LSM, liver stiffness measurement; LT, liver transplantation; MASLD, metabolic dysfunction-associated steatotic liver disease; N/A, not applicable
